# New approaches of green silver nanoparticles for cancer and biomedical applications: a review

**DOI:** 10.37349/etat.2025.1002341

**Published:** 2025-10-23

**Authors:** Puja Kumari, Khushi Quadri, Renu Kadian, Saloni Mishra, Aafrin Waziri, Kaustub Jumle, Kumar Sambhav Verma, Md Sabir Alam

**Affiliations:** University of Valladolid, Spain; ^1^Dhanrua School of Nursing & Paramedics, At- Awadhara, Pavery, Dhanrua, Patna 804451, BR, India; ^2^Department of Pharmaceutics, School of Pharmaceutical Education and Research, Jamia Hamdard, New Delhi 110062, DL, India; **^3^** Ram Gopal College of Pharmacy, Sultanpur, Gurugram 122506, HR, India; ^4^SGT School of Pharmacy, SGT University, Chandu, Budhera, Gurugram 122505, HR, India; ^5^University School of Biotechnology, Guru Gobind Singh Indraprastha University, Dwarka, Delhi 110078, DL, India; ^6^Amity Institute of Biotechnology, Amity University Rajasthan, Jaipur 303002, RJ, India

**Keywords:** green synthesis, silver nanoparticles, cancer therapy, biomedical applications, nanotechnology

## Abstract

The green synthesis of silver nanoparticles (AgNPs) has recently gained prominence as a sustainable and eco-friendly alternative to conventional physical and chemical methods. Utilizing biological entities such as plant extracts, bacteria, fungi, and biomolecules, the method acts by both reducing and stabilizing mechanisms. It does not use any harmful chemical substances, thus proving to be eco-friendly. Green-synthesized AgNPs exhibit enhanced biocompatibility, stability, and targeted delivery of the drug due to the use of naturally derived surface capping agents. These unique characteristics allow selective interference with cancer cells. The mechanism involved is the generation of reactive oxygen species (ROS), the induction of apoptosis, DNA damage, and cell cycle arrest. Green AgNPs also possess broad-spectrum antimicrobial, catalytic, antiparasitic, and anti-inflammatory properties, supporting the fact that they can be utilised in biomedical fields such as drug delivery, bioimaging, biosensing, tissue engineering, and regenerative medicine. Recent advancements have focused on controlling NP size, shape, and surface functionality to maximize efficacy while simultaneously minimizing cytotoxicity. This review provides a comprehensive analysis of the latest green synthesis strategies, their characterizations, and the molecular mechanisms by which they exert anticancer effects. Recent patents highlight the clinical potential of AgNPs in cancer therapy. US Patent 12201650 (2025) describes green synthesis using *Caralluma sinaica*, while other patents (WO2007001453, US7462753) outline adaptable biomedical formulations. Studies on biogenic AgNPs also show significant tumor inhibition and selective cytotoxicity against cancer cells. Furthermore, the article discusses current biomedical applications and critically evaluates the limitations, such as reproducibility, toxicity concerns, and scalability for clinical translation. Addressing these challenges is essential for the integration of green AgNPs into mainstream cancer therapeutics. The convergence of nanotechnology and biologically derived synthesis opens promising avenues for the development of safe, effective, and environmentally sustainable medical innovations.

## Introduction

Nanotechnology is now recognized as one of the critical research endeavors of the early 21st century. This field attracted more interest at the beginning of the 21st century, and scientists have taken advantage of the unique features of atomic and molecular assemblages produced at nanometer scales [1]. Richard Zsigmondy first proposed the concept of a “nanometer” and was awarded the Nobel Prize for this in chemistry in 1925. He studied nanomaterials and then characterized their particle size, shape, and morphology with the help of a microscope. Nanoparticles (NPs) are the most essential components for the development of nanostructures. NPs are regulated by Newton’s laws of motion, and quantum mechanics shows that subjects are larger than an atom or simple molecules [2]. A technique that is applied at the nanoscale is called nanotechnology and has unique phenomena, making it suitable for different applications. Its size ranges from 1 to 100 nm of matter at the atomic and molecular scale [3, 4]. Compared to materials with a larger scale, they have different properties. Nanomaterials have been used in different physical and chemical methods to achieve novel commercial applications, and societal benefits are also possible. At the end of the 20th century, new openings were sought for the development of innovative nanomaterials and nanosystems. The novel discovery is of nanoscale materials, processes, and phenomena, as well as new experimental and theoretical study methods. This field is enhancing scientific and technological possibilities [5]. Nanotechnology encompasses the usage of nanomaterials, as well as many methods for synthesizing, such as physical, chemical, and biological, at scales ranging from a single atom or molecule to submicron dimensions. Similar effects on society and the economy were seen in the 20th century with the development of semiconductors, information, and cellular and molecular biology technologies [6]. Nanotechnology has the potential to have a significant impact on the synthesis of novel materials for the development of new products, the replacement of existing manufacturing equipment, the reformulation of novel materials and chemicals for improved performance, and the use of novel materials and chemicals for the remediation of the environment [7]. Normally, bioentities such as enzymes, amino acids, dietary fibers, RNA, DNA, and viruses occur naturally as components of biological structures, but nanotechnology can be used to synthesize, mimic, or manipulate them for various applications [8].

### Metallic NPs (MNPs)

In the field of nanotechnology, MNPs were able to show a variety of properties and have demonstrated various novel opportunities in the field of NPs. The presence of suitable functional groups differentiates MNPs. They can be synthesized and modified to bind with medications, antibodies, and ligands [9]. MNPs play a significant role because they have the potential to be used in new fields of nanoscience and technology [10, 11]. Many researchers have shown that MNPs can be synthesized using biological sources such as algae, fungi, and bacteria, as well as metals like gold (Au), silver (Ag), titanium (Ti), cadmium (Cd), iron (Fe), zinc (Zn), and magnesium (Mg), among others, for diverse biomedical and industrial applications [12].

### Silver NPs

Silver NPs (AgNPs) exhibit a wide array of biological activities, including anti-inflammatory, antiseptic, and pro-healing effects, making them ideal for healthcare applications like wound dressings, medical coatings, and surgical instruments. They also find use in cosmetics, food packaging, and textile industries [13–15].

Silver and its compounds have recently gained renewed attention in microbiology, medicine, and biomedicine due to their broad-spectrum antimicrobial potential. AgNPs are particularly valued for their antibacterial properties and are widely used in medical applications such as catheters, dental procedures, and burn treatments [16–18]. Compared to silver ions (Ag⁺), AgNPs offer similar antimicrobial efficacy while mitigating side effects associated with ionic silver or silver nitrate [19]. Their mode of action, primarily involving direct interaction with bacterial membranes, allows them to overcome many traditional antibiotic resistance mechanisms [20]. Their antimicrobial effect is influenced by their small size and high surface area, which enable better interaction with microbial cells and gradual Ag⁺ release under biological conditions [21].

AgNPs are synthesized through various techniques, including chemical, photochemical, electrochemical, and green methods. Among them, chemical reduction using agents like sodium citrate and borohydride is common, though it poses toxicity concerns [22–24]. Green synthesis has emerged as a safer, eco-friendly alternative using plant extracts, microbes, and natural polymers [25]. Additionally, AgNPs are employed in bio and electrochemical sensors due to their excellent catalytic and electronic properties [26, 27]. While their antibacterial potential is well-documented, uncertainties remain regarding their toxicity and mechanisms at the cellular level [28]. These NPs can interfere with cellular proteins, nucleic acids, and membranes, causing microbial death, but their safety as an antibiotic substitute is still debated [29, 30].

#### Comparative advantages and disadvantages of AgNPs over other nanocarriers

##### Advantages

AgNPs have garnered significant attention as nanocarriers due to their unique physicochemical and biological properties. Compared to other metallic and polymeric nanocarriers, AgNPs exhibit intrinsic antimicrobial, anticancer, anti-inflammatory, and antioxidant activities, making them suitable for multifunctional biomedical applications without the need for additional active agents [31, 32]. Their ability to generate reactive oxygen species (ROS) and induce apoptosis in cancer cells provides a dual benefit of acting as a therapeutic agent and a carrier simultaneously [33, 34].

Unlike liposomes or polymeric NPs, AgNPs possess a high surface area-to-volume ratio and strong surface plasmon resonance effects, allowing for enhanced drug loading, targeted delivery, and optical tracking capabilities [35, 36]. Their surface can be readily functionalized with a wide range of biomolecules, targeting ligands, or polymers to enhance biocompatibility and specificity [37, 38]. Green synthesis methods, in particular, offer eco-friendly, cost-effective, and scalable routes to fabricate AgNPs with enhanced biocompatibility compared to chemically synthesized ones [39, 40].

##### Disadvantages

However, AgNPs also present several limitations when compared to other nanocarriers. One of the major concerns is their potential cytotoxicity, which is largely dose-, size-, and shape-dependent [41, 42]. In contrast, liposomes and biodegradable polymeric NPs such as poly lactic-co-glycolic acid (PLGA) often show better in vivo biocompatibility and reduced immune clearance rates [43, 44]. Moreover, the long-term toxicity, accumulation in vital organs, and lack of uniform regulatory guidelines for AgNPs pose challenges for clinical translation [45, 46].

In addition, while AgNPs exhibit strong antimicrobial activity, this can also disrupt normal microbiota if not carefully targeted, unlike more specific nanocarrier systems [47]. Furthermore, their stability in physiological environments can be limited, requiring stabilizers or surface coatings to maintain functionality, which adds complexity to their design [48, 49]. On the other hand, polymeric micelles and dendrimers offer controlled release profiles and pH-responsive behaviour, which are areas where AgNPs may require further optimization [50, 51].

Despite these challenges, ongoing research is directed toward combining AgNPs with other nanocarriers (e.g., core-shell systems or hybrid nanosystems) to mitigate toxicity while leveraging their therapeutic benefits [52, 53]. Advances in biofunctionalization, green synthesis, and targeting strategies are expected to improve the clinical viability of AgNP-based nanocarriers in the future [54–56]. In summary, while AgNPs hold distinct advantages in multifunctionality and simplicity, careful engineering and safety assessment are essential to address their limitations and establish them as competitive alternatives to conventional nanocarriers [57–65].

### Synthesis of MNPs

**Figure 1:**
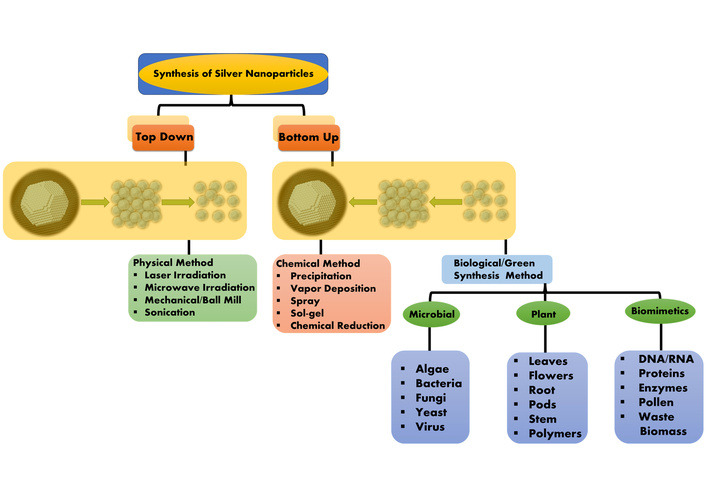
Synthesis of metallic nanoparticles.

Two alternative methods can be used for the synthesis of MNPs, namely (i) the top-down approach and (ii) the bottom-up approach [64, 65]. The “top-down” method is based on building structures from the parts of much bigger devices by monolithic processing, which is more readily possible with current technology. Consumer electronics semiconductor devices have demonstrated the usefulness of this technique, as well as the “bottom-up” method, which involves the methodical self-assembly of molecules, atoms, or other fundamental building blocks of matter to create device structures [66]. Furthermore, the synthesis of NPs involves the use of three distinct techniques: physical, chemical, and biological processes Figure 1.

Physical methods use top-down techniques, whereas chemical and biological methods use bottom-up techniques for the synthesis of MNPs. Several chemical and physical processes are used to synthesize MNPs that try to regulate the physical characteristics of the particles. Most of these technologies are used in early stages of development, and issues with NP preparation stability, crystal growth control, toxicity, and particle aggregation are common [67–70].

#### Physical methods for NPs synthesis (top-down approach)

Multiple techniques are used in the physical synthesis of NPs, including vapor phase: Arc discharge, hydrogen plasma, laser pyrolysis, and chemical vapor condensation. Solid phase: Ball mill [71]. The high quantity of energy requirement is one of the main drawbacks associated with these approaches, as well as the substantial period needed to complete the entire process (for example, costly, limited manufacturing rate, enormous energy consumption for maintaining high pressure and temperature) [72].

#### Chemical methods for NP synthesis (bottom-up approach)

The chemical substances that are most frequently used in the synthesis of MNPs include chemical reductants: Alcohol, molecular hydrogen, hydrazine, sodium tetrahydroborate, citrate, *N*,*N*-dimethyl formamide, polyols, ethylene glycol, and cyclodextrin are some of the ingredients in lithium aluminum hydrate. Sources of energy: Light, ultraviolet/visible light, electricity, heat, sonochemical energy, and X-rays are examples of photo energy. However, they are not considered green chemical reagents due to their potential environmental harm. Sodium borohydride is a well-known reducing agent that has numerous applications in chemical synthesis. As a result, it has been widely utilized to convert metal salts into NPs. The Brust-Schiffrin two-phase technique is one of the most widely used synthesis processes [73]. Chemical reduction occurs at an oil-water interface, followed by thiolated molecule adsorption and stabilization in the organic phase. This method has been widely used because of its simplicity and efficiency in expanding the understanding and applications of gold and AgNPs [74]*.* The use of sodium borohydride and colloidal stabilization is combined with capping molecules. Although occasionally mild compounds are used, such as derivatives of β-cyclodextrin [75] or clays [76], Citrate ions are used as both reducing and stabilizing agents, which is a common technique for the synthesis of spherical Au and AgNPs [77]. Carboxylic acids, polymers, aromatic and halogenated organic compounds, as well as surfactants, have been characterized as capping molecules and are relevant to the development of MNPs that have various morphologies [78, 79].

#### Green/Biological synthesis of NPs (bottom-up approach)

Nowadays, for the production of NPs, the use of biological synthesis has become a popular alternative to conventional techniques. Biosynthesis involves using unicellular and multicellular organisms such as actinomycetes [80], bacteria [81], fungi [82], plants [83], viruses [84], and yeast [85], entities in an environment-friendly green chemistry-based method. It is a non-toxic and eco-friendly method of NPs formation with a wide range of shapes, sizes, compositions, and physicochemical properties utilizing living organisms [86]. Green or biological NP synthesis prevents toxicity by using low pressure, temperature, and pH at a substantially lower cost [87]. Alkaloids, proteins, flavonoids, reducing sugars, polyphenols, and other compounds that are present in biomaterials work as capping and reducing agent for NPs from their metal salt predecessors [88]. Initial confirmation of the reduction of the metal salt precursor to its eventual NPs is aided by visualizing the color shift in the colloidal solution. Recently, several organisms, including unicellular and multicellular, have been employed for the green synthesis of NPs. The biological elements, including primary and secondary metabolites, perform as catalysts to promote metal ion reduction and the development of MNPs. On the surface of MNPs, these same reducing agents or other molecules may form a stabilizing layer, preventing or at least decreasing the capacity to assemble or become disordered throughout the production process [89]. Additionally, the production of MNPs made via biological methods can be influenced by experimental conditions such as temperature, pH, and reagent concentration [90].

##### Green synthesis of MNPs by plants

Green synthesis of MNPs can utilize organisms from all biological kingdoms. Fortunately, a lot of these creatures that are suitable for green synthesis are also species that contribute to biodiversity and are raised for food and feed. Researchers investigating the green synthesis of MNPs were the first to choose plants because of their large biomass, variety of species availability [91]. These chemicals are the same responsible for the plant's status as a significant bioreactor and molecular supplier, also used in green synthesis methods [92]. In reality, it is now generally acknowledged that plants produce several metabolites that can interact to stabilize the surface of MNPs and/or transform metal ions into their metallic equivalents [93]. Amino acids are thought to be the main compounds that cause plants to reduce metal ions [94], citric acid [95], flavonoids [96], phenolic compounds [97], terpenoids [98], tannins [99], enzymes [100], peptides [101], saponins [102], polysaccharides [103], heterocyclic compounds [104], among others. The green synthesis of MNPs, which is mediated by plants, utilizes entire organisms as well as organ and tissue extracts [105]. It uses different parts of the plant, such as root, leaves, seeds, barks, fruits, and others, which may create nano-objects with a variety of features [106]*.* Therefore, they ought to be taken into account separately. Depending on the requirements of each part of the plant and the types of abiotic or biotic stress that a plant may be exposed to, each plant component has a distinct phytochemical profile with a different composition or concentration.

##### NP synthesis using fungi

Extracellular synthesis of MNPs, such as AgNPs, utilizing fungi is also a promising option due to their cost-effective, large-scale manufacturing. Fungal strains are preferred over bacterial species due to their higher tolerance and metal-bioaccumulation ability [107]. It has been shown that the fungus *Fusarium oxysporum* is capable of synthesizing AgNPs with diameters ranging from 5 to 15 nm that have been stabilized by fungal protein capping. The fungus *Fusarium oxysporum* can produce NPs outside of cells [108]. This study reported the intracellular production of Ag, Au, cadmium sulphide (CdS), lead sulphide (PbS), molybdenum sulphide (MoS), and zinc sulphide (ZnS) NPs [109]. Fungi have certain advantages over bacteria when it comes to producing NPs, including simpler scaling up and downstream processing, better economics, and a bigger surface area offered by fungal mycelia [110]. Although the rate of synthesis of NPs should rise due to the increased amount of proteins released by fungi, quality is compromised as some fungi are Phytopathogenic and may pose a threat to human health [111].

##### NP synthesis using algae

Algae, which could be used to produce MNPs naturally, have been determined to accumulate heavy metals. Algae, a wide range of aquatic microorganisms, have been extensively employed to synthesize AgNPs, and their sizes range from microscopic to macroscopic (Rhodophyta). *Chlorella vulgaris* is a type of unicellular algae that can develop NPs in a variety of shapes, including tetrahedral, decahedral, and icosahedral particles that gather close to the surface [112]. Algal extractʼs proteins, in particular, function as a stabilizing, reducing, and shape-controlling agent [113]. The AuNPsʼ actual yield, kinetics, and colloidal stability were studied in micro-algal cells of *Euglena gracilis* grown in mixotrophic (exposed to light and produced in a culture medium enriched with organic carbon) or autotrophic conditions [114].

##### Bacterial-mediated NPs synthesis

Bacteria are typically used for NPs synthesis due to the low conditions required, ease of purification, and high yield. As a result, bacteria have become the most widely studied microorganism, receiving the title of “the factory of nanomaterials”. *Bacillus thuringiensis* has recently been applied to synthesize AgNPs with sizes ranging from 43.52 to 142.97 nm [115]. Bacteria can be utilized as a biocatalyst for the production of inorganic materials, as a bioscaffold for mineralization, or as an active participant in NPs synthesis [116]. Bacteria can synthesize nanomaterials in broth media as extracellular or intracellular during an incubation period. Because of this feature, bacterial biosynthesis of NPs is a reasonable, versatile, and acceptable technology for large-scale manufacturing.

##### Polymer-mediated MNPs

Various polymers were used to synthesize AgNPs, such as Gum Acacia [117], Gum Arabic [118], Chitosan/Guar gum/Gum Ghatti [119], Tara Gum [120], Aloe Vera [121], κ-carrageenan [122], for different biomedical applications.

### Characterization techniques

NPs are synthesized by shrinking their size through physical or chemical methods [123]. Importantly, processing frequently introduces imperfections on the surface because the shape, size, and surface structure of NPs are heavily dependent on each other. These surface defects can have a major impact on the overall surface and physicochemical characteristics [124].

**Figure 2:**
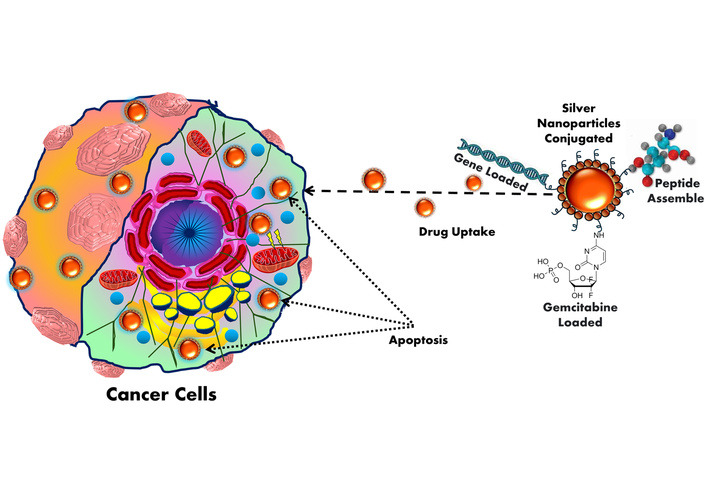
Characterization of metallic nanoparticles.

The NPs are characterized by using a variety of techniques to determine factors like size distribution, particle size, shape, and surface area. These are especially important if homogeneous NPs characterization is required for a specific application. Numerous common spectroscopy and microscopy methods are used to characterize NPs, including UV-visible spectroscopy (UV-Vis), X-ray diffraction (XRD), Fourier transform infrared spectroscopy (FTIR), dynamic light scattering (DLS), atomic force microscope (AFM), transmission electron microscopy (TEM), scanning electron microscopy (SEM), energy dispersive X-ray (EDX), and Raman spectroscopy, which are all common spectroscopy and microscopy techniques. These techniques, based on microscopy, are considered direct methods used for acquiring data from images of NPs, and are widely utilized to determine the size and morphological properties of NPs (see Figure 2) [125–127].

#### UV-Vis spectrophotometry

UV-Vis is the most used method to characterize MNPs [128]. When synthesized from its specific metal, it produces a distinctive peak with significant visible-range absorptions [129]. The surface plasmon response (SPR) peak is well known for a range of MNPs, ranging in size, and various synthesized NPs have demonstrated that it is ideal for characterizing particles in the absorption band with a wavelength of about 200–800 nm [130]. In AgNPs, the valence and conduction bands are very close together. These bands provide a SPR absorption band by enabling unlimited electron migration. Particle size, dielectric medium, and the chemical environment all have an impact on how well AgNPs are absorbed. The stability of biologically generated AgNPs was studied for almost a year, and a SPR peak at the same wavelength was found using UV-Vis spectrophotometry [131].

#### XRD analysis

XRD analysis methods are used for analyzing the structure of NPs, where MNPs show amorphous and crystalline nature, identified with the help of X-rays, which can penetrate deeply into the material [132]. The formation of crystalline NPs is verified by the diffraction pattern [133].

The Debye-Scherrer equation is used to quantify particle size from XRD data by estimating the width of the Bragg reflection peak according to the equation [134].

$$t=\frac{k\lambda }{\beta cos\theta }$$

Where, t = Crystallite size, k = shape factor, λ = wavelength of the radiation, θ = Bragg’s angle, β = full width at half maximum.

To explore the structural characteristics of many materials, including polymers, glasses, biomolecules, and superconductors, XRD can be used. Additionally, XRD is an effective technique for researching nanomaterials [135].

#### FTIR

FTIR can be used to analyze different capping agents, the involvement of biomolecules in the synthesis of MNPs, and the surface chemistry of synthesized MNPs [136]. In FTIR, the sample transmits photons; some of them are absorbed by the sample, and the rest pass through it. The resulting spectra show the transmission and absorption properties of the sample material [137]. It is affordable, appropriate, simple, and non-intrusive to evaluate the role of biological molecules in the conversion of silver nitrate to silver [138].

#### TEM

TEM is a particularly significant tool for characterizing by giving detailed information about their shape, size, internal morphology, and crystallographic structure [139]. TEM operates by transmitting a beam of electrons through an ultra-thin specimen; the interactions between the electrons and the atoms in the sample generate high-resolution images. Compared to SEM, TEM offers nearly 1,000 times higher resolution, allowing visualization at the atomic or molecular scale [140]. This makes it especially useful for observing the internal lattice structure, defects, and particle dispersion in nanomaterials. Additionally, TEM images yield more precise insights into the crystallinity, orientation, and morphological variations of NPs, making it an essential technique in nanoscience and biomedical research [141].

#### DLS

The particle size and its size distribution can be determined widely using the technique of DLS. In DLS parameters, zeta sizer and zeta potential have been used to describe NPs frequently and have been used to gauge the size. Additionally, DLS is also widely used to size MNPs in liquid form [142]. Its role in characterizing distinct types of NPs has been established. Because of Brownian motion, the size of NPs obtained through DLS is often larger than that determined by TEM; it is possible to estimate the average size of NPs in liquids using this technique [143].

#### AFM

In 1986, Binning, Quate, and Gerber developed the technology of the AFM to improve upon the drawbacks of the scanning tunnelling microscope (STM) [144]. AFM can provide three-dimensional (3D) topographic images with nanoscale resolution, and it makes the most efficient approach for morphological and structural investigation of polymeric nanocomposites under AFM [145]. The most significant development in AFM has been its ability to assess non-conductive samples' surface topography at sub-nanometer resolution [146]. Additionally, the AFM is useful since it requires less sample preparation and may be utilized in fields of natural settings. The sample does not need to be conductive or metallized before being subjected to morphological analysis. This characteristic method is an extraordinary tool for the direct characterization of a variety of samples with complex morphological structures. By moving a tip attached to a flexible cantilever across the sample surface, an atomic-scale measurement of surface morphology is accomplished using AFM. The deflection of cantilevers during scanning is used to determine the force acting between the tip and the sample [147].

### MNPs in cancer therapy

MNPs have gained significant attention in cancer therapy due to their exceptional physicochemical properties. These include a high surface-area-to-volume ratio, ease of functionalization, and the ability to penetrate biological membranes efficiently [148]. MNPs are typically composed of elements like gold (Au), silver (Ag), iron oxide (Fe₃O₄), and platinum (Pt), each offering unique characteristics suited for biomedical applications [149–157]. In cancer treatment, MNPs are being utilized for various purposes such as targeted drug delivery, photothermal therapy, tumor imaging, and immunotherapy. Their nanoscale size allows them to circulate through the bloodstream and accumulate preferentially in tumor tissues, thereby offering an advanced strategy to overcome the limitations of conventional chemotherapy [158].

#### MNPs for tumor targeting

One of the most important advantages of MNPs is their ability to selectively accumulate in tumor tissues while sparing normal, healthy cells. This property not only increases the efficacy of the therapy but also minimizes systemic toxicity and side effects associated with non-specific drug distribution. Tumor targeting by MNPs occurs through two fundamental mechanisms: passive targeting and active targeting [159]. Passive targeting exploits the abnormal architecture of tumor blood vessels, thus enhancing permeability and retention (EPR) effect. Tumors generally have leaky vasculature and inefficient lymphatic drainage, which allow NPs to passively accumulate in the tumor interstitial space over time. This forms the basic foundation for NPs-mediated drug delivery systems [160]. Active targeting, on the other hand, takes tumor specificity a step further by modifying the surface of NPs with specific ligands such as antibodies, aptamers, peptides, or small molecules. These ligands recognize and bind to receptors that are overexpressed on the surface of cancer cells, ensuring that the therapeutic agent is delivered precisely where it is needed. This strategy improves drug localization, enhances cellular uptake, and boosts the therapeutic index [161].

#### Targeting mechanisms and surface functionalization of MNPs

The surface of MNPs can be engineered to enhance their functionality and compatibility with the biological environment. Surface modification not only prolongs the circulation time of NPs in the bloodstream but also facilitates their recognition and binding to target cells. One of the most used stabilizing agents is polyethylene glycol (PEG), which helps to reduce immune system recognition and opsonization by serum proteins, thereby enhancing their half-life [162]. Further functionalization involves conjugating targeting moieties that bind selectively to tumor-associated receptors. Among these, the matrix metalloproteinase-2 (MMP-2) receptor has been a notable focus. MMP-2 is an enzyme overexpressed in many invasive and metastatic tumors. NPs functionalized with MMP-2-sensitive peptides can undergo enzyme-mediated degradation, releasing their therapeutic payload precisely in the tumor microenvironment (TME) where MMP-2 activity is elevated [163]. Another popular targeting strategy involves the folate receptor, which is abundantly expressed in a range of cancers, including breast, ovarian, and lung cancers. Folic acid, a small molecule with high affinity for the folate receptor, can be conjugated to the surface of MNPs to achieve receptor-mediated endocytosis into tumor cells. This method is particularly beneficial because folate is a vitamin that does not trigger immunogenic responses, making it a safe and effective targeting ligand [164]. HER2/neu, a receptor tyrosine kinase commonly found in aggressive breast cancers, is another important biomarker for targeted therapy. NPs can be functionalized with monoclonal antibodies such as trastuzumab to selectively target HER2-positive tumors. These antibody-coated MNPs can carry chemotherapeutic drugs or photosensitizers to the tumor site and, in the case of AuNPs, can even be used for photothermal ablation by converting light into heat, thereby killing cancer cells [165].

#### Passive and active targeting of MNPs

Targeting strategies using MNPs can be broadly divided into basic (passive) and active approaches. As discussed, basic targeting utilizes the natural tendency of NPs to accumulate in tumor tissues due to the EPR effect. While this method improves drug delivery compared to systemic administration, it does not provide the level of precision required to completely spare healthy tissues [166]. Active MNPs, in contrast, are designed to respond to internal or external stimuli for controlled drug release. These stimuli can include pH changes, redox gradients, enzyme activity, temperature shifts, or the application of external magnetic fields. For example, pH-sensitive AuNPs release their drug payload only in the acidic environment typical of tumors, thus minimizing off-target effects. Similarly, MNPs such as iron oxide (Fe₃O₄) can be guided to tumor sites using external magnets and can also be heated under alternating magnetic fields for hyperthermia-based cancer therapy [167]. These advanced MNPs offer multiple levels of control, combining targeting, therapy, and real-time imaging into a single theranostics platform.

#### Cancer immunotherapy using MNPs

Beyond direct targeting of cancer cells, MNPs are playing a transformative role in the field of cancer immunotherapy, which seeks to harness the body’s immune system to identify and destroy cancer cells. MNPs can serve as delivery vehicles for a wide range of immunomodulatory agents such as cytokines, immune checkpoint inhibitors, and tumor-associated antigens [168]. One of the promising applications involves using NPs as cancer vaccines. AuNPs, for instance, can be loaded with tumor antigens and adjuvants to activate dendritic cells, which in turn prime T-cells to recognize and kill cancer cells. This approach shows potential in generating a strong and long-lasting anti-tumor immune response [169]. Iron oxide NPs are also gaining traction in immunotherapy. They can be taken up by macrophages and help polarize them from the M2 (tumor-supporting) phenotype to the M1 (tumor-fighting) phenotype. By reprogramming the TEM, these MNPs reduce immunosuppression and facilitate the infiltration and activity of cytotoxic immune cells [170]. Additionally, MNPs can be engineered to block immune checkpoint pathways, such as PD-1/PD-L1 and CTLA-4, either by delivering antibodies or RNA-based inhibitors directly into tumor sites, thereby restoring immune function and enabling T-cells to eliminate cancer cells more effectively in Table 1 [171].

**Table 1:** Overview of cancer types and experimental models in AgNPs-based anticancer studies.

**Plant source**	**Characterization**	**In vitro model**	**Mechanism**	**References**
*Pinus roxburghii*	UV-Vis, FTIR, XRD, EDX, SAED, FESEM, and HRTEM	Lung adenocarcinomas (A549), prostatic small cell carcinomas (PC-3)	Apoptosis via mitochondrial depolarization, DNA damage, ROS, cell cycle arrest, and caspase-3 activation	[172]
*Phyllanthus emblica*	UV-Vis, TEM, FTIR, SEM-EDX, XRD, DLS-Zeta potential, TGA, and HRTEM	Lung cancer cell line (A549)	Elevated ROS levels, enhanced DNA damage, and cell death	[173]
*Cynara scolymus* (Artichoke)	UV-Vis, FTIR, SEM, DLS, and EDX	Breast cancer cells (MCF-7)	Reduce cell migration, expression of Bax, and suppression of Bcl-2	[174]
*Moringa oleifera*	XRD, FTIR, HRTEM, EDX, and PL	In-vitro cytotoxicity and cell viability of human cancer cell HT-29	Induce apoptosis	[175]
*Tamarindus indica*	UV-Vis, FTIR, EDS, SEM, and TEM	MCF-7 human breast cancer cell line	Induce apoptosis	[176]
*Achillea biebersteinii*	UV-Vis, FTIR, TEM, DLS, and EDX	MCF-7 human breast cancer cell line	Triggered apoptosis through caspase activation and modulation of Bax and Bcl-2 expression	[177]
*Punica granatum*	UV-Vis, FTIR, DLS, EDX, SEM, and XRD	Human cervical cancer cells (HeLa)	Reduce cell viability	[178]
*Gloriosa superba*	UV-Vis, HRTEM, EDX, DLS, and XRD	MCF-7 cell line	High cytotoxicity due to interactions with cellular proteins and DNA, leading to cell death	[179]
*Teucrium polium*	UV-Vis, FTIR, SEM, and XRD	MNK45 human gastric cancer cell line	Cytotoxic activity induces apoptosis	[180]
*Melia dubia*	UV-Vis, XRD, EDS, and SEM	Human breast cancer (KB) cell line	Show activity against the KB cell line	[181]
*Ulva lactuca*	UV-Vis, FTIR, TEM, and EDX	Human colon cancer HCT-116 cells	Higher levels of P53, Bax, and P21, along with lower Bcl-2, point to cell death driven by p53-related apoptosis	[182]
*Cucumis prophetarum*	UV-Vis, FTIR, DLS, XRD, SEM, and EDX	A549, MDA-MB-231, hepatocellular carcinoma (HepG2), and MCF-7 cell line	Antiproliferative potential against selected cancer cell lines	[183]
*Rosa damascena*	UV-Vis, FTIR, DLS, SEM, HRTEM, XRD, and EDX	Human lung adenocarcinoma (A549)	Inducing apoptosis, generating ROS, and disrupting mitochondrial membrane potential lead to cell death	[184]
*Gossypium hirsutum*	UV-Vis, FTIR, LS, SEM, TEM, and XRD	Human lung cancer cells (A549)	Activate apoptosis in cancer cells by mitochondria-mediated pathways	[185]
*Syzygium aromaticum*	UV-Vis, HRTEM, and EDX	MCF-7 breast and A549 lung cell lines	Induced apoptosis via oxidative stress mechanisms	[186]
*Podophyllum hexandrum*	TEM, XRD, and FTIR	Human cervical cancer cell line (HeLa)	Decrease cell proliferation, increase intracellular ROS, DNA damage, and apoptosis	[187]
*Heliotropium indicum*	SEM, EDX	HeLa cervical cancer cell line	Inhibits cell growth in a dose and time-dependent manner	[188]
*Azadirachta indica*	FTIR, TEM, and DLS	MCF-7 and HeLa cell lines; in vivo model (Balb/C mice)	Alter pro-inflammatory cytokine levels and pro-apoptotic protein expressions	[189]
Gum arabic	UV-Vis, TEM	Oral tongue squamous cell carcinoma (CAL-127 cells)	Inhibits hypoxia through its suppressive effect on the HIF-1α protein, and its regulators miR-210 and miR-21	[190]
*Alternanthera sessilis*	UV-Vis, EDX, SAED, FTIR, HRTEM, and AFM	Cervical cancer cell line (HeLa)	Induce apoptosis	[191]

AgNPs: silver nanoparticles; UV-Vis: UV-visible spectroscopy; FTIR: Fourier transform infrared spectroscopy; XRD: X-ray diffraction; EDX: energy dispersive X-ray; SAED: selected area electron diffraction; FESEM: field emission scanning electron microscopy; HRTEM: high-resolution transmission electron microscopy; TEM: transmission electron microscopy; TGA: thermogravimetric analysis; SEM: scanning electron microscopy; DLS: dynamic light scattering; PL: photoluminescence; EDS: energy dispersive X-ray spectroscopy; LS: light scattering; AFM: atomic force microscope; ROS: reactive oxygen species; HIF-1α: hypoxia-inducible factor 1-alpha.

#### Mechanistic approach of MNPs for tumor targeting

**Figure 3:**
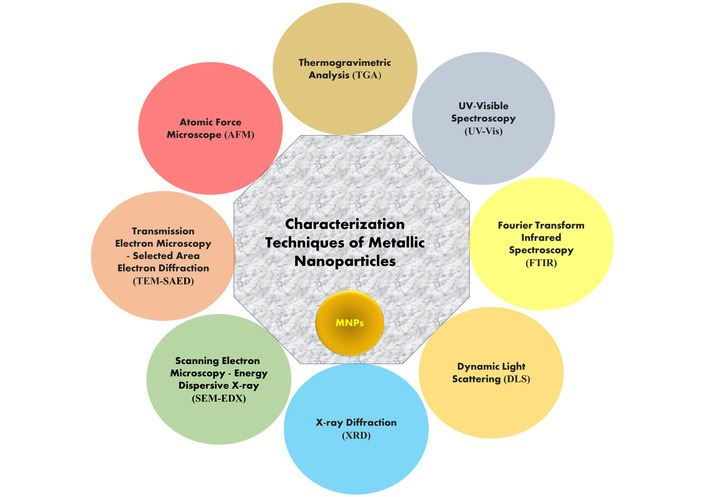
Cancer treatment by silver nanoparticles.

MNPs, including AuNPs, AgNPs, iron oxide, zinc oxide, and copper oxide, exhibit multifaceted mechanisms for tumor-specific targeting and theranostics [192]. Engineered with precise size, shape, and surface chemistry, MNPs exploit the EPR effect for passive accumulation in tumor tissues due to aberrant vasculature and impaired lymphatic drainage. Smaller particle sizes further improve tumor penetration and therapeutic efficacy in cancer [193]. Particle size plays a critical role in the anticancer efficacy of NPs, as smaller particles exhibit greater cellular uptake and deeper tumor penetration. Studies have shown that NPs below 50 nm induce higher levels of apoptosis in cancer cells due to enhanced ROS generation and DNA damage. Thus, reducing particle size significantly improves the therapeutic potential of nanocarriers against cancer [194]. For active targeting, their surfaces are functionalized with monoclonal antibodies, peptides, or aptamers that selectively bind overexpressed tumor-associated antigens or receptors [195]. AuNPs, in particular, are utilized in photothermal therapy owing to their strong surface plasmon resonance in the NIR region, enabling efficient photo-induced hyperthermia and tumor ablation (see Figure 3) [196]. AgNPs exhibit potent cytotoxicity via redox imbalance, mitochondrial dysfunction, and DNA damage through excessive ROS production [197]. Superparamagnetic iron oxide NPs (SPIONPs) allow magnetic field-guided delivery, real-time MRI tracking, and local hyperthermia induction [198]. Other MNPs like ZnO and CuO trigger endosomal escape and initiate intrinsic apoptotic cascades by disrupting redox homeostasis [199]. Additionally, MNPs serve as nanocarriers for chemotherapeutics, siRNA, or CRISPR systems, enabling TME-responsive, site-specific delivery to minimize systemic exposure [200]. Functionalization with pH- or enzyme-sensitive linkers ensures stimuli-triggered release within acidic or protease-rich TMEs. MNPs further enhance imaging modalities such as MRI, CT, and photoacoustic imaging, facilitating image-guided therapy [201]. These integrated diagnostic and therapeutic capabilities position MNPs as next-generation nanotheranostic platforms for precision oncology [202].

Figure 3 shows AgNPs functionalized with peptides, genes, or chemotherapeutic drugs like gemcitabine for targeted cancer therapy. Exploiting the EPR effect, AgNPs accumulate in tumor tissues and are internalized by cancer cells. This leads to efficient drug uptake and induction of apoptosis, enhancing anticancer efficacy.

##### Patents

Several patents and recent studies highlight the potential of AgNPs in cancer therapy. A recent patent describes the green synthesis of AgNPs using *Caralluma sinaica*, offering biocompatible particles with potential anticancer activity [203]. Patent reviews on nanotheranostic silver systems emphasize their dual role in imaging and therapy, enabling targeted tumor treatment [204]. In vivo studies on biogenic silver/silver chloride NPs demonstrated significant inhibition of Ehrlich ascites carcinoma in mice, improving survival by approximately 75% [205]. Foundational patents, such as WO2007001453 and US7462753, cover synthesis and biomedical formulations of AgNPs, forming the basis for anticancer adaptations. Additionally, plant-derived AgNPs reported in recent literature show selective cytotoxicity toward cancer cells while sparing normal tissues, reinforcing their promise as eco-friendly and effective anticancer agents [206, 207].

### Biomedical applications of AgNPs

#### Antibacterial activity of AgNPs

**Figure 4:**
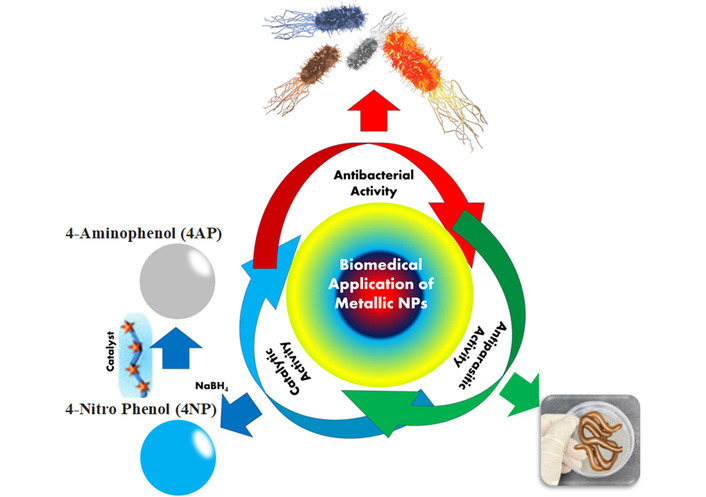
Biomedical applications of silver nanoparticles.

In the current scenario, plants are used to synthesize AgNPs. It is simple to synthesize using plant extracts or even the entire plant [207, 208]. In the health sector, AgNPs are frequently used as antibacterial agents, for food preservation, textile coatings, and with significant environmental applications (see Table 2) [208, 209]. AgNPs are used against antibacterial activity, the ability of AgNPs to reduce silver ions, to more frequently attach to thiol groups in bacterial proteins, interrupting their physiological activity, and causing cell death. According to many researchers, AgNPs penetrate and then destroy the bacterial membrane, preventing proper cell function, which causes structural damage and finally cell death, as shown in Figure 4 [210].

**Table 2:** Antibacterial activity of AgNPs.

**Polymer type**	**Characterization**	**Application**	**Reference**
Sodium alginate	UV-Vis, TEM, and XRD	Antibacterial activity against Gram-negative and Gram-positive bacteria	[211]
Pine gum	SPR, EDX, FTIR, TEM, and XRD	Against odor- or skin infection-causing bacteria, also *Brevibacterium linens*	[212]
Gum ghatti	UV-Vis, TEM, and XRD	The AgNPs can be easily integrated for a variety of biological applications (both Gram-positive and Gram-negative)	[213]
Chitosan/Guar gum/Gum ghatti	UV-Vis, XRD, and SEM	Due to the synergistic interaction of AgNPs used against *Staphylococcus aureus* and *Escherichia coli* bacteria suggested promising antibacterial efficacy	[214]
Piyar gum	UV-Vis, FTIR, DLS, SEM, TEM, and AFM	Against both Gram-negative bacterial strains, i.e., *Escherichia coli* and *Avibacterium avium*	[215]
Neem gum	UV-Vis, FTIR, TEM, and AFM	Antibacterial activity against clinical isolates of *Salmonella enteritidis* and *Bacillus cereus*	[216]
*Aloe barbadensis* Miller	UV-Vis	Antibacterial activity against Gram-negative and Gram-positive bacteria	[217]
Starch-gelatin	UV-Vis, TEM, SEM, XRD, and thermal method	Antibacterial activity against Gram-negative and Gram-positive bacteria	[218]

AgNPs: silver nanoparticles; UV-Vis: UV-visible spectroscopy; TEM: transmission electron microscopy; XRD: X-ray diffraction; SPR: surface plasmon response; EDX: energy dispersive X-ray; FTIR: Fourier transform infrared spectroscopy; SEM: scanning electron microscopy; DLS: dynamic light scattering; AFM: atomic force microscope.

#### Catalytic activity of AgNPs

In chemistry and materials science, the creation of reliable, recyclable, environmentally friendly catalysts is considered an enormous challenge. Understanding this fieldʼs potency, while using MNPs, is now important due to the fieldʼs reliable development. More importantly, the creation of biodegradable, reusable catalysts helps to reduce the amount of waste that must be disposed of, and these catalysts are seen as essential [219–221]. The significance of environmental protection for humans has increased in recent years, and some poisonous dye molecules, like Methylene orange, Methylene blue, Congo red, 4-nitrophenol, and eosin Y, are hazardous to the environment. Hazardous dyes can be used to reduce smaller organic molecules and non-toxic species by reductants like NaBH_4_; however, the rate of reduction is particularly slow (see Table 3). High reactivity, as well as the particular surface area of AgNPs, can accelerate the reduction of dyes, improving the efficiency of the reduction process [222].

**Table 3:** Catalytic activity of silver nanoparticles.

**Polymer**	**Characterization**	**Application**	**References**
Salvia officinalis leaf extract	UV-Vis, FTIR, DLS, SEM, TEM, EDS, and TGA	Against toxic dye shows significant catalytic activity in the degradation of CR dye.	[223]
Gum acacia	UV-Vis, FTIR, TEM, and XRD	Used against toxic dye (4NP to 4AP).	[224]
Gum arabic	UV-Vis, TEM, and SWV	The technology was used to find MB in samples of river Water since its ability to recover values was beneficial.	[225]
Acacia nilotica gum extract	UV-Vis, FTIR, TEM, and XRD	Studies have been carried out into the reduction 4NP to 4AP by NaBH_4_ (reducing agents) catalyzed using AgNPs.	[226]
Chitin	FTIR, XRD, XPS, SEM, and TGA	Used to 4NP reduced to 4AP in catalyst activity.	[227]
Crocus haussknechtii extract	UV-Vis, FTIR, XRD, and SEM	The degradation of a Congo Red dye was used to evaluate the catalytic activity of synthesized NPs in the presence of NaBH_4_.	[228]
Trigonella foenum-graecum seeds	UV-Vis, FTIR, and XRD	Used against toxic dye such as hazardous dyes, methyl orange, methylene blue and eosin Y.	[229]

UV-Vis: UV-visible spectroscopy; FTIR: Fourier transform infrared spectroscopy; DLS: dynamic light scattering; SEM: scanning electron microscopy; TEM: transmission electron microscopy; EDS: energy dispersive X-ray spectroscopy; TGA: thermogravimetric analysis; XRD: X-ray diffraction; SWV: square wave voltammetry; XPS: X-ray photoelectron spectroscopy; 4NP: 4-nitro phenol; 4AP: 4-aminophenol; AgNPs: silver nanoparticles.

#### Anti-parasitic activity of AgNPs

Leishmaniasis is a parasitic disease caused by parasites of the genus *Leishmania* [230]. AgNPs were found to exhibit larvicidal action against sandfly bites in Table 4. The current scenario causes concern due to the costly nature and limited supply of antileishmanial medications, as well as the development of resistance to these drugs. However, due to the formation of ROS, this parasite is extremely sensitive to AgNPs. Under UV light, NPs have a combinatory detrimental impact on *Leishmania tropica* [231–233].

**Table 4:** Anti-parasitic activity of silver nanoparticles.

**Polymer**	**Characterization**	**Application**	**Reference**
Ginger extract	UV-Vis spectroscopy, MTT test, TEM	*Leishmania major*ʼs in vitro promastigotes and amastigote forms are positively impacted	[234]
*Fusarium oxysporum*	UV-Vis, TEM	Promastigotes and amastigote forms were used in in vivo investigations against *Leishmania amazonensis* as a possible treatment for American Cutaneous Leishmaniasis (ACL)	[235]
Chitosan	UV-Vis, FTIR, DLS, AFM, and TEM. Resazurin and MTT colorimetric tests	More active against *Leishmania amazonensis*	[236, 237]

UV-Vis: UV-visible spectroscopy; MTT: 3-(4,5-dimethylthiazol-2-yl)-2,5-diphenyltetrazolium bromide; TEM: transmission electron microscopy; FTIR: Fourier transform infrared spectroscopy; DLS: dynamic light scattering; AFM: atomic force microscope.

## Discussion

Recent advancements in the green synthesis of AgNPs have led to a paradigm shift in biomedical applications, with particular emphasis on their potent anticancer, antimicrobial, catalytic, and oxidative stress-inducing properties. Numerous studies demonstrate that biologically synthesized AgNPs, using plant extracts, bacteria, fungi, and algae, possess enhanced bioactivity and safety profiles compared to their chemically synthesized counterparts. In cancer-related research, green AgNPs have shown promising cytotoxic effects against a wide range of human cancer cell lines, including breast, lung, liver, cervical, and colorectal cancers. These NPs selectively induce apoptosis in cancer cells while sparing healthy cells, primarily through mitochondrial disruption, overproduction of ROS, and activation of intrinsic apoptotic pathways such as caspase-3 and -9. Furthermore, they interfere with key cell signaling mechanisms like PI3K/Akt and MAPK, leading to reduced cell viability, DNA fragmentation, and inhibition of cell proliferation. In the microbial domain, green AgNPs have demonstrated significant inhibitory effects against both Gram-positive and Gram-negative bacteria, including multidrug-resistant strains like *Staphylococcus aureus*, *Escherichia coli*, and *Pseudomonas aeruginosa*. Their antimicrobial action is mainly attributed to disruption of microbial membranes, oxidative stress induction, and binding with microbial DNA and proteins, ultimately leading to cell death. From a catalytic perspective, green AgNPs have exhibited efficient activity in degrading organic dyes and environmental pollutants under mild conditions, suggesting their dual utility in biomedical and environmental domains. The natural phytochemicals involved in their synthesis provide a stabilizing shell, enhancing electron transfer capabilities and improving NP dispersion, which contributes to their catalytic efficiency. Additionally, the oxidative stress-inducing nature of green AgNPs plays a central role in both cancer and antimicrobial mechanisms, as controlled ROS generation leads to oxidative damage in targeted cells without affecting surrounding healthy tissues when dosed appropriately. These findings support the multifunctionality of green AgNPs and highlight their role as oxidative mediators, selective cytotoxic agents, and efficient nano-catalysts. However, variability in synthesis conditions, such as source material, temperature, pH, and reaction time, can lead to differences in size, shape, and surface charge of the NPs, which in turn affect their biological performance. As a result, a major challenge remains in the standardization and reproducibility of green synthesis protocols. Moreover, although in vitro and some in vivo studies have confirmed the therapeutic potential of green AgNPs, further investigation is needed to evaluate their long-term toxicity, pharmacokinetics, and biodistribution in human systems. Safety and regulatory concerns also pose limitations to their clinical translation. Nonetheless, the integration of nanotechnology with sustainable biosynthesis techniques presents a viable and promising approach for developing next-generation therapeutic agents. With continued interdisciplinary research and optimization of synthesis strategies, green AgNPs hold significant promise as effective tools for cancer treatment, antimicrobial interventions, catalytic applications, and oxidative therapeutics, offering a multifaceted platform for future medical and biotechnological innovations.

## Conclusions

MNPs offer a highly promising platform for integrating diagnostics and therapy—an emerging approach known as “theranostics”. By fusing targeting, therapeutic, and imaging capabilities into a single nano-system, MNPs are paving the way for personalized and precision medicine in oncology. Among them, AgNPs stand out due to their strong antimicrobial, anticancer, and anti-inflammatory properties, as well as their ease of functionalization. However, several critical challenges remain. These include concerns related to toxicity, long-term colloidal and physiological stability, immune system clearance, and regulatory approval pathways. To overcome these hurdles, ongoing research is focused on enhancing the biocompatibility, specificity, and biodegradability of MNPs through surface modifications, green synthesis techniques, and targeted ligand conjugation. Successful clinical translation of these technologies also demands standardized manufacturing protocols, batch-to-batch consistency, and rigorous preclinical safety evaluations. The ability to combine multiple therapeutic strategies—such as immunotherapy, photothermal therapy, gene delivery, and chemotherapy—within a single nanoplatform opens exciting avenues for next-generation cancer treatments. With continued innovation and collaboration across disciplines, AgNPs and other MNPs are expected to play a pivotal role in the evolution of integrated and individualized cancer therapy.

## Abbreviations

AFM: atomic force microscope
AgNPs: silver nanoparticles
DLS: dynamic light scattering
EPR: enhancing permeability and retention
FTIR: Fourier transform infrared spectroscopy
MMP-2: matrix metalloproteinase-2
MNPs: metallic nanoparticles
NPs: nanoparticles
ROS: reactive oxygen species
SEM: scanning electron microscopy
SPR: surface plasmon response
TEM: transmission electron microscopy
TME: tumor microenvironment
XRD: X-ray diffraction

## Declarations

### Copyright

© The Author(s) 2025.

## References

[1] Shahzadi S, Fatima S, Ain QU, Shafiq Z, Janjua MRSA (2025). A review on green synthesis of silver nanoparticles (SNPs) using plant extracts: a multifaceted approach in photocatalysis, environmental remediation, and biomedicine. RSC Adv.

[2] Harun-Ur-Rashid M, Foyez T, Krishna SBN, Poda S, Imran AB (2025). Recent advances of silver nanoparticle-based polymer nanocomposites for biomedical applications. RSC Adv.

[3] Sati A, Ranade TN, Mali SN, Yasin HKA, Pratap A (2025). Silver Nanoparticles (AgNPs): Comprehensive Insights into Bio/Synthesis, Key Influencing Factors, Multifaceted Applications, and Toxicity-A 2024 Update. ACS Omega.

[4] Ahmad A, Haneef M, Ahmad N, Kamal A, Jaswani S, Khan F (2024). Biological synthesis of silver nanoparticles and their medical applications (Review). World Acad Sci J.

[5] Rupanshi, Kumar V, Yadav N, Singh D, Beniwal V, Chhabra J (2025). Biogenic Silver Nanoparticles as Next-Generation Green Catalysts for Multifaceted Applications. Trans Tianjin Univ.

[6] Li XQ, Xie SL, Xie HD, Shen MQ, Ma ZH, Liu H (2024). Exploring biomedical applications with silver and copper nanotechnology. cMat.

[7] Magar A, Dharashive V, Shafi S, Kartale G, Bedare S, Bhosale V (2024). Silver Nanoparticles: A Modern Era of Nanotechnology. Asian J Pharm Res Dev.

[8] Falke PB, Shelke PG, Hatwar PR, Bakal RL, Kohale NB (2024). A comprehensive review on Nanoparticle: Characterization, classification, synthesis method, silver nanoparticles and its applications. GSC Biol Pharm Sci.

[9] Gherasim O, Puiu RA, Bîrcă AC, Burdușel A, Grumezescu AM (2020). An Updated Review on Silver Nanoparticles in Biomedicine. Nanomaterials (Basel).

[10] Bhosale VS, Dharashive V, Londhe GD, Sagale KA, Natkar AS, Magar AV (2025). Nanoparticulate Mucoadhesive System: Innovative Approach in Drug Delivery. Asian J Pharm Res Dev.

[11] Kaehler T (1994). Nanotechnology: basic concepts and definitions. Clin Chem.

[12] Horikoshi S, Serpone N Introduction to Nanoparticles.

[13] Roco MC, Williams RS, Alivisatos P Nanotechnology Research Directions: IWGN Workshop Report.

[14] Prasad SR, Elango K, Damayanthi D, Saranya JS (2013). Formulation and Evaluation of Azathioprine Loaded Silver Nanopartilces for The Treatment of Rheumatoid Arthritis. Asian J Biomed Pharm Sci.

[15] Li L, Hu J, Yang W, Alivisatos AP (2001). Band Gap Variation of Size- and Shape-Controlled Colloidal CdSe Quantum Rods. Am Chem Soc.

[16] Bhushan B, the Author (2017). Introduction to Nanotechnology. Springer Handbook of Nanotechnology.

[17] Roco MC (1999). Towards a US National Nanotechnology Initiative. J Nanopart Res.

[18] Bhushan B (2015). Governance, policy, and legislation of nanotechnology: a perspective. Microsyst Technol.

[19] Bhushan B, Winkelmann K, Bhushan B Introduction to Nanotechnology: History, Status, and Importance of Nanoscience and Nanotechnology Education. Global Perspectives of Nanoscience and Engineering Education.

[20] Lee J, Mahendra S, Alvarez PJJ (2010). Nanomaterials in the construction industry: a review of their applications and environmental health and safety considerations. ACS Nano.

[21] Chang SS, Shih CW, Chen CD, Lai WC, Wang CRC (1999). The Shape Transition of Gold Nanorods. Am Chem Soc.

[22] Shnoudeh AJ, Hamad I, Abdo RW, Qadumii L, Jaber AY, Surchi HS, Tekade RK Synthesis, Characterization, and Applications of Metal Nanoparticles. Biomaterials and Bionanotechnology.

[23] Konop M, Damps T, Misicka A, Rudnicka L (2016). Certain Aspects of Silver and Silver Nanoparticles in Wound Care: A Minireview. J Nanomater.

[24] Barillo DJ, Marx DE (2014). Silver in medicine: a brief history BC 335 to present. Burns.

[25] Clement JL, Jarrett PS (1994). Antibacterial silver. Met Based Drugs.

[26] Alexander JW (2009). History of the medical use of silver. Surg Infect (Larchmt).

[27] Medici S, Peana M, Nurchi VM, Zoroddu MA (2019). Medical Uses of Silver: History, Myths, and Scientific Evidence. J Med Chem.

[28] Javed MN, Alam MS, Pottoo FH (2015). Metallic nanoparticle alone and/or in combination as novel agent for the treatment of uncontrolled electric conductance related disorders and/or seizure, epilepsy & convulsions.

[29] Pandit J, Bharti C, Gupta S, Munawar SM, Sabjan KB, Quadri K, Mohamed WMY A new era of nanotechnology applied in neurological disease treatments. Essential Guide to Neurodegenerative Disorders.

[30] Quadri K, Kadian R, Thakur S, Chaturvedi S, Rawat G, Waziri A, Mohamed WMY Potential role of probiotics for neurological disease treatment. Essential Guide to Neurodegenerative Disorders.

[31] You C, Han C, Wang X, Zheng Y, Li Q, Hu X (2012). The progress of silver nanoparticles in the antibacterial mechanism, clinical application and cytotoxicity. Mol Biol Rep.

[32] Castellano JJ, Shafii SM, Ko F, Donate G, Wright TE, Mannari RJ (2007). Comparative evaluation of silver-containing antimicrobial dressings and drugs. Int Wound J.

[33] Chen X, Schluesener HJ (2008). Nanosilver: a nanoproduct in medical application. Toxicol Lett.

[34] Fong J (2005). The Use of Silver Products in the Management of Burn Wounds: Change in Practice for the Burn Unit at Royal Perth Hospital. Primary Intention: Aust J Wound Manage.

[35] Sunilbhai CA, Alam, MS, Sadasivuni, KK, Ansari JR, Sadasivuni KK, Cabibihan JJ, A M Al-Ali AK, Malik RA (2022). SPR Assisted Diabetes Detection. Advanced Bioscience and Biosystems for Detection and Management of Diabetes.

[36] White RJ (2001). An historical overview of the use of silver in wound management. Br J Nurs.

[37] Faunce T, Watal A (2010). Nanosilver and global public health: international regulatory issues. Nanomedicine (Lond).

[38] Barillo DJ, Pozza M, Margaret-Brandt M (2014). A literature review of the military uses of silver-nylon dressings with emphasis on wartime operations. Burns.

[39] Abboud EC, Settle JC, Legare TB, Marcet JE, Barillo DJ, Sanchez JE (2014). Silver-based dressings for the reduction of surgical site infection: review of current experience and recommendation for future studies. Burns.

[40] Bates MN (2006). Mercury amalgam dental fillings: an epidemiologic assessment. Int J Hyg Environ Health.

[41] Xu Y, Gao C, Li X, He Y, Zhou L, Pang G (2013). In vitro antifungal activity of silver nanoparticles against ocular pathogenic filamentous fungi. J Ocul Pharmacol Ther.

[42] Oyanedel-Craver VA, Smith JA (2008). Sustainable colloidal-silver-impregnated ceramic filter for point-of-use water treatment. Environ Sci Technol.

[43] Bandyopadhyaya R, Sivaiah MV, Shankar PA (2008). Silver-embedded granular activated carbon as an antibacterial medium for water purification. J Chem Technol Biotechnol.

[44] Bhandari M, Raj S, Alam MS (2025). Recent innovations in nanomedicine and nano-based techniques for the treatment of breast cancer. Bioimpacts.

[45] Dowsett C (2004). The use of silver-based dressings in wound care. Nurs Stand.

[46] Murthy AB, Palaniappan V, Chandramohan A, Narasimhan M (2025). Silver in dermatology - From ancient use to modern innovations. JSSTD.

[47] Lansdown ABG (2006). Silver in health care: antimicrobial effects and safety in use. Curr Probl Dermatol.

[48] Kadian R, Pandit J, Bharti C, Rabiya, Waziri A, Kumari P Application of MNPs in Targeted Delivery and Genetic Manipulations.

[49] Hooda N, Ahlawat A, Kumari P, Alam S, Ansari JR (2023). Role of Nanomedicine for Targeted Drug Delivery in Livestock: Future Prospective. Pharm Nanotechnol.

[50] Vermeulen H, van Hattem JM, Storm-Versloot MN, Ubbink DT (2007). Topical silver for treating infected wounds. Cochrane Database Syst Rev.

[51] Lansdown ABG Silver in Healthcare: Its Antimicrobial Efficacy and Safety in Use.

[52] Maillard J, Hartemann P (2013). Silver as an antimicrobial: facts and gaps in knowledge. Crit Rev Microbiol.

[53] Klasen HJ (2000). A historical review of the use of silver in the treatment of burns. II. Renewed interest for silver. Burns.

[54] Silver S, Phung LT (1996). Bacterial heavy metal resistance: new surprises. Annu Rev Microbiol.

[55] Slawson RM, Van Dyke MI, Lee H, Trevors JT (1992). Germanium and silver resistance, accumulation, and toxicity in microorganisms. Plasmid.

[56] Abou El-Nour KM, Eftaiha A, Al-Warthan A, Ammar RA (2010). Synthesis and applications of silver nanoparticles. Arab J Chem.

[57] Murali Mohan Y, Lee K, Premkumar T, Geckeler KE (2007). Hydrogel networks as nanoreactors: A novel approach to silver nanoparticles for antibacterial applications. Polymer.

[58] Ahamed M, Alsalhi MS, Siddiqui MKJ (2010). Silver nanoparticle applications and human health. Clin Chim Acta.

[59] Graham C (2005). The role of silver in wound healing. Br J Nurs.

[60] Beyth N, Houri-Haddad Y, Domb A, Khan W, Hazan R (2015). Alternative antimicrobial approach: nano-antimicrobial materials. Evid Based Complement Alternat Med.

[61] Hemmati S, Rashtiani A, Zangeneh MM, Mohammadi P, Zangeneh A, Veisi H (2019). Green synthesis and characterization of silver nanoparticles using Fritillaria flower extract and their antibacterial activity against some human pathogens. Polyhedron.

[62] Zhang Z, Shen W, Xue J, Liu Y, Liu Y, Yan P (2018). Recent advances in synthetic methods and applications of silver nanostructures. Nanoscale Res Lett.

[63] Lee SH, Jun B (2019). Silver Nanoparticles: Synthesis and Application for Nanomedicine. Int J Mol Sci.

[64] Tarannum N, Divya, Gautam YK (2019). Facile green synthesis and applications of silver nanoparticles: a state-of-the-art review. RSC Adv.

[65] Beyene HD, Werkneh AA, Bezabh HK, Ambaye TG (2017). Synthesis paradigm and applications of silver nanoparticles (AgNPs), a review. Sustainable Mater Technol.

[66] Miralles-Comins S, Zanatta M, Sans V (2022). Advanced Formulations Based on Poly(ionic liquid) Materials for Additive Manufacturing. Polymers (Basel).

[67] Al-Thabaiti SA, Al-Nowaiser FM, Obaid AY, Al-Youbi AO, Khan Z (2008). Formation and characterization of surfactant stabilized silver nanoparticles: a kinetic study. Colloids Surf B Biointerfaces.

[68] Banerjee J, Narendhirakanan RT (2011). Biosynthesis of silver nanoparticles from Syzygium cumini (L.) seed extract and evaluation of their in vitro antioxidant activities. Dig J Nanomater Biostruct.

[69] Li B, Zhong WH (2011). Review on polymer/graphite nanoplatelet nanocomposites. J Mater Sci.

[70] Ashour AA, Raafat D, El-Gowelli HM, El-Kamel AH (2015). Green synthesis of silver nanoparticles using cranberry powder aqueous extract: characterization and antimicrobial properties. Int J Nanomedicine.

[71] Banala RR, Nagati VB, Karnati PR (2015). Green synthesis and characterization of Carica papaya leaf extract coated silver nanoparticles through X-ray diffraction, electron microscopy and evaluation of bactericidal properties. Saudi J Biol Sci.

[72] Chernousova S, Epple M (2013). Silver as antibacterial agent: ion, nanoparticle, and metal. Angew Chem Int Ed Engl.

[73] Stensberg MC, Wei Q, McLamore ES, Porterfield DM, Wei A, Sepúlveda MS (2011). Toxicological studies on silver nanoparticles: challenges and opportunities in assessment, monitoring and imaging. Nanomedicine (Lond).

[74] Das RK, Pachapur VL, Lonappan L, Naghdi M, Pulicharla R, Maiti S (2017). Biological synthesis of metallic nanoparticles: plants, animals and microbial aspects. Nanotechnol Environ Eng.

[75] Thakkar KN, Mhatre SS, Parikh RY (2010). Biological synthesis of metallic nanoparticles. Nanomedicine.

[76] Mijatovic D, Eijkel JCT, Berg Avd (2005). Technologies for nanofluidic systems: top-down vs. bottom-up--a review. Lab Chip.

[77] Brust M, Kiely CJ (2002). Some recent advances in nanostructure preparation from gold and silver particles: a short topical review. Colloids Surf A Physicochem Eng Asp.

[78] Kowshik M, Ashtaputre S, Kharrazi S, Vogel W, Urban J, Kulkarni SK (2002). Extracellular synthesis of silver nanoparticles by a silver-tolerant yeast strain MKY3. Nanotechnol.

[79] Huang H, Yang X (2005). One-step, shape control synthesis of gold nanoparticles stabilized by 3-thiopheneacetic acid. Colloids Surf A Physicochem Eng Asp.

[80] Mandal S, Phadtare S, Sastry M (2005). Interfacing biology with nanoparticles. Curr Appl Phys.

[81] Sharma G, Pandey S, Ghatak S, Watal G, Rai PK, Tripathi DK, Ahmad P, Sharma S, Chauhan DK, Dubey NK Potential of Spectroscopic Techniques in the Characterization of “Green Nanomaterials”. Nanomaterials in Plants, Algae, and Microorganisms.

[82] Pantidos N, Horsfall LE (2014). Biological Synthesis of Metallic Nanoparticles by Bacteria, Fungi and Plants. J Nanomed Nanotechnol.

[83] Brust M, Walker M, Bethell D, Schiffrin DJ, Whyman R (1994). Synthesis of thiol-derivatised gold nanoparticles in a two-phase Liquid–Liquid system. J Chem Soc, Chem Commun.

[84] Liz-Marzán LM (2013). Gold nanoparticle research before and after the Brust-Schiffrin method. Chem Commun (Camb).

[85] Male KB, Li J, Bun CC, Ng SC, Luong JHT (2008). Synthesis and Stability of Fluorescent Gold Nanoparticles by Sodium Borohydride in the Presence of Mono-6-deoxy-6-pyridinium-β-cyclodextrin Chloride. J Phys Chem C.

[86] Liz-Marzan LM, Philipse AP (1995). Stable hydrosols of metallic and bimetallic nanoparticles immobilized on imogolite fibers. J Phys Chem.

[87] Turkevich J, Stevenson PC, Hillier J (1951). A study of the nucleation and growth processes in the synthesis of colloidal gold. Discuss Faraday Soc.

[88] Grzelczak M, Pérez-Juste J, Mulvaney P, Liz-Marzán LM (2008). Shape control in gold nanoparticle synthesis. Chem Soc Rev.

[89] Lohse SE, Burrows ND, Scarabelli L, Liz-Marzán LM, Murphy CJ (2014). Anisotropic Noble Metal Nanocrystal Growth: The Role of Halides. Chem Mater.

[90] Ahmad A, Senapati S, Khan MI, Kumar R, Sastry M (2003). Extracellular Biosynthesis of Monodisperse Gold Nanoparticles by a Novel Extremophilic Actinomycete, *Thermomonospora* sp. Langmuir.

[91] Klaus-Joerger T, Joerger R, Olsson E, Granqvist C (2001). Bacteria as workers in the living factory: metal-accumulating bacteria and their potential for materials science. Trends Biotechnol.

[92] Mukherjee P, Ahmad A, Mandal D, Senapati S, Sainkar SR, Khan MI (2001). Fungus-Mediated Synthesis of Silver Nanoparticles and Their Immobilization in the Mycelial Matrix: A Novel Biological Approach to Nanoparticle Synthesis. Nano Letters.

[93] Kumar P, Singh P, Kumari K, Mozumdar S, Chandra R (2011). A green approach for the synthesis of gold nanotriangles using aqueous leaf extract of *Callistemon viminalis*. Mater Lett.

[94] Merzlyak A, Lee S (2006). Phage as templates for hybrid materials and mediators for nanomaterial synthesis. Curr Opin Chem Biol.

[95] Rahi S, Pandit J, Quadri K, Bharti C, Alam MS, Jain A, Mody N, Palakurthi S Vesicular carriers for stimuli-responsive drug delivery to tumors: design considerations. Tumor-Targeting with Stimuli-Responsive Vesicular Nanocarriers.

[96] Mohanpuria P, Rana NK, Yadav SK (2008). Biosynthesis of nanoparticles: technological concepts and future applications. J Nanopart Res.

[97] Fouda A, Mohmed A, Elgamal MS, EL-Din Hassan S, Salem SS, Shaheen TI (2017). Facile Approach towards Medical Textiles via Myco-synthesis of Silver Nanoparticles. Der Pharma Chem.

[98] Kuppusamy P, Yusoff MM, Maniam GP, Govindan N (2016). Biosynthesis of metallic nanoparticles using plant derivatives and their new avenues in pharmacological applications - An updated report. Saudi Pharm J.

[99] Iravani S (2011). Green synthesis of metal nanoparticles using plants. Green Chem.

[100] Bonatto CC, Silva LP (2014). Higher temperatures speed up the growth and control the size and optoelectrical properties of silver nanoparticles greenly synthesized by cashew nutshells. Ind Crops Prod.

[101] Chokkareddy R, Redhi GG, Kanchi S, Ahmed S Green Synthesis of Metal Nanoparticles and its Reaction Mechanisms. Green Metal Nanoparticles.

[102] Jha AK, Prasad K, Prasad K, Kulkarni AR (2009). Plant system: natureʼs nanofactory. Colloids Surf B Biointerfaces.

[103] Jeevanandam J, Chan YS, Danquah MK (2016). Biosynthesis of Metal and Metal Oxide Nanoparticles. ChemBioEng Rev.

[104] Shao Y, Jin Y, Dong S (2004). Synthesis of gold nanoplates by aspartate reduction of gold chloride. Chem Commun (Camb).

[105] Vickers NJ (2017). Animal Communication: When I'm Calling You, Will You Answer Too?. Curr Biol.

[106] Shankar SS, Ahmad A, Pasrichaa R, Sastry M (2003). Bioreduction of chloroaurate ions by geranium leaves and its endophytic fungus yields gold nanoparticles of different shapes. J Mater Chem.

[107] Sivaraman SK, Elango I, Kumar S, Santhanam V (2009). A green protocol for room temperature synthesis of silver nanoparticles in seconds. Curr Sci.

[108] Kisimba K, Krishnan A, Faya M, Byanga K, Kasumbwe K, Vijayakumar K (2023). Synthesis of Metallic Nanoparticles Based on Green Chemistry and Their Medical Biochemical Applications: Synthesis of Metallic Nanoparticles. J Renewable Mater.

[109] Li Q, Mahendra S, Lyon DY, Brunet L, Liga MV, Li D (2008). Antimicrobial nanomaterials for water disinfection and microbial control: potential applications and implications. Water Res.

[110] Narayanan KB, Sakthivel N (2011). Green synthesis of biogenic metal nanoparticles by terrestrial and aquatic phototrophic and heterotrophic eukaryotes and biocompatible agents. Adv Colloid Interface Sci.

[111] Tan YN, Lee JY, Wang DIC (2010). Uncovering the design rules for peptide synthesis of metal nanoparticles. J Am Chem Soc.

[112] Park Y, Hong YN, Weyers A, Kim YS, Linhardt RJ (2011). Polysaccharides and phytochemicals: a natural reservoir for the green synthesis of gold and silver nanoparticles. IET Nanobiotechnol.

[113] McCullen SD, Stevens DR, Roberts WA, Clarke LI, Bernacki SH, Gorga RE (2007). Characterization of electrospun nanocomposite scaffolds and biocompatibility with adipose-derived human mesenchymal stem cells. Int J Nanomedicine.

[114] Huang X, Wu H, Pu S, Zhang W, Liao X, Shi B (2011). One-step room-temperature synthesis of Au@Pd core–shell nanoparticles with tunable structure using plant tannin as reductant and stabilizer. Green Chem.

[115] Marchiol L (2012). Synthesis of Metal Nanoparticles in Living Plants. Ital J Agron.

[116] Kharissova OV, Dias HVR, Kharisov BI, Pérez BO, Pérez VMJ (2013). The greener synthesis of nanoparticles. Trends Biotechnol.

[117] Prasad R, Pandey R, Barman I (2016). Engineering tailored nanoparticles with microbes: *quo vadis*?. Wiley Interdiscip Rev Nanomed Nanobiotechnol.

[118] Rai M, Yadav A, Gade A (2009). Silver nanoparticles as a new generation of antimicrobials. Biotechnol Adv.

[119] Velamakanni RP, Gothalwal R, Velamakanni RS, Ayinampudi SR, Vuppugalla P, Merugu R, Srivastava M, Malik MA, Mishra PK (2022). Fungi-Mediated Green Synthesis of Nanoparticles and Their Renewable Energy Applications. Green Nano Solution for Bioenergy Production Enhancement.

[120] Alani F, Moo-Young M, Anderson W (2012). Biosynthesis of silver nanoparticles by a new strain of Streptomyces sp. compared with Aspergillus fumigatus. World J Microbiol Biotechnol.

[121] Basavaraja S, Balaji SD, Lagashetty A, Rajasab AH, Venkataraman A (2008). Extracellular biosynthesis of silver nanoparticles using the fungus *Fusarium semitectum*. Mater Res Bull.

[122] Minuto A, Spadaro D, Garibaldi A, Gullino ML (2006). Control of soilborne pathogens of tomato using a commercial formulation of Streptomyces griseoviridis and solarization. Crop Protection.

[123] Luangpipat T, Beattie IR, Chisti Y, Haverkamp RG (2011). Gold nanoparticles produced in a microalga. J Nanopart Res.

[124] Xie J, Lee JY, Wang DIC, Ting YP (2007). Silver nanoplates: from biological to biomimetic synthesis. ACS Nano.

[125] Dahoumane SA, Yéprémian C, Djédiat C, Couté A, Fiévet F, Coradin T (2016). Improvement of kinetics, yield, and colloidal stability of biogenic gold nanoparticles using living cells of *Euglena gracilis* microalga. J Nanopart Res.

[126] Banu AN, Balasubramanian C (2014). Optimization and synthesis of silver nanoparticles using Isaria fumosorosea against human vector mosquitoes. Parasitol Res.

[127] Srivastava S, Bhargava A, Pathak N, Srivastava P (2019). Production, characterization and antibacterial activity of silver nanoparticles produced by Fusarium oxysporum and monitoring of protein-ligand interaction through in-silico approaches. Microb Pathog.

[128] Virk K, Sharma K, Kapil S, Kumar V, Sharma V, Pandey S (2022). Synthesis of gum acacia-silver nanoparticles based hydrogel composites and their comparative anti-bacterial activity. J Polym Res.

[129] Sharma K, Majhi S, Tripathi CSP, Guin D (2022). Electrochemical Sensing Platform based on Greenly Synthesized Gum Arabic Stabilized Silver Nanoparticles for Hydrogen Peroxide and Glucose. J Electrochem Soc.

[130] Bhat VG, Masti SP, Narasagoudr SS, Chougale RB, Kumar P, Vantamuri AB (2023). Development and characterization of Chitosan/Guar gum /Gum ghatti bionanocomposites with in situ silver nanoparticles. Chem Data Collect.

[131] de Oliveira Bianchi JR, Menezes de Souza S, Boggione Santos IJ (2023). Post-Harvest Application of Tara Gum Coating Incorporated With Silver Nanoparticles for Preservation of Banana. Biointerface Res Appl Chem.

[132] Naveen KV, Saravanakumar K, Sathiyaseelan A, Wang MH (2022). Eco-friendly synthesis and characterization of *Aloe vera*/Gum Arabic/silver nanocomposites and their antibacterial, antibiofilm, and wound healing properties. Colloid Interface Sci Commun.

[133] You S, Zhang X, Wang Y, Jin Y, Wei M, Wang X (2022). Development of highly stable color indicator films based on κ-carrageenan, silver nanoparticle and red grape skin anthocyanin for marine fish freshness assessment. Int J Biol Macromol.

[134] Sepeur S Nanotechnology: Technical Basics and Applications.

[135] Meyers MA, Mishra A, Benson DJ (2006). Mechanical properties of nanocrystalline materials. Prog Mater Sci.

[136] Poinern GEJ (2015). A Laboratory Course in Nanoscience and Nanotechnology.

[137] Eppler AS, Rupprechter G, Anderson EA, Somorjai GA (2000). Thermal and Chemical Stability and Adhesion Strength of Pt Nanoparticle Arrays Supported on Silica Studied by Transmission Electron Microscopy and Atomic Force Microscopy. J Phys Chem B.

[138] Fedlheim DL, Foss CA (2001). Metal Nanoparticles: Synthesis, Characterization, and Applications.

[139] Sastry M, Patil V, Sainkar SR (1998). Electrostatically Controlled Diffusion of Carboxylic Acid Derivatized Silver Colloidal Particles in Thermally Evaporated Fatty Amine Films. J Phys Chem B.

[140] Karikalan N, Karthik R, Chen S, Velmurugan M, Karuppiah C (2016). Electrochemical properties of the acetaminophen on the screen printed carbon electrode towards the high performance practical sensor applications. J Colloid Interface Sci.

[141] Rahaie M, Naghavi MR, Alizadeh H, Malboobi MA, Dimitrov K (2011). A novel DNA-based nanostructure for single molecule detection purposes. Int J Nanotechnol.

[142] Zhang X, Liu Z, Shen W, Gurunathan S (2016). Silver Nanoparticles: Synthesis, Characterization, Properties, Applications, and Therapeutic Approaches. Int J Mol Sci.

[143] Laksaci H, Khelifi A, Belhamdi B, Trari M (2017). Valorization of coffee grounds into activated carbon using physic—chemical activation by KOH/CO2. J Environ Chem Eng.

[144] Clament Sagaya Selvam N, Kumar RT, Kennedy LJ, Vijaya JJ (2011). Comparative study of microwave and conventional methods for the preparation and optical properties of novel MgO-micro and nano-structures. J Alloys Compd.

[145] Nii T, Ishii F (2005). Dialkylphosphatidylcholine and egg yolk lecithin for emulsification of various triglycerides. Colloids Surf B Biointerfaces.

[146] Giannini C, Ladisa M, Altamura D, Siliqi D, Sibillano T, De Caro L (2016). X-ray Diffraction: A Powerful Technique for the Multiple-Length-Scale Structural Analysis of Nanomaterials. Crystals.

[147] Rajeshkumar S, Bharath LV (2017). Mechanism of plant-mediated synthesis of silver nanoparticles - A review on biomolecules involved, characterisation and antibacterial activity. Chem Biol Interact.

[148] Rohman A, Che Man YB (2010). Fourier transform infrared (FTIR) spectroscopy for analysis of extra virgin olive oil adulterated with palm oil. Food Res Int.

[149] Shanmuganathan R, Karuppusamy I, Saravanan M, Muthukumar H, Ponnuchamy K, Ramkumar VS (2019). Synthesis of Silver Nanoparticles and their Biomedical Applications - A Comprehensive Review. Curr Pharm Des.

[150] Noruzi M (2015). Biosynthesis of gold nanoparticles using plant extracts. Bioprocess Biosyst Eng.

[151] Pandit J, Alam MS, Ansari JR, Singhal M, Gupta N, Waziri A Multifaced Applications of Nanoparticles in Biological Science.

[152] Alam MS, Javed MN, Ansari JR (2023). Metallic Nanoparticles for Health and the Environment.

[153] Alam MS, Garg M, Bhalla V, Kumari P, Kadyan R, Anjum MM Trends in Theranostic Applications of Metallic Nanoparticles.

[154] Devi L, Ansari TM, Alam MS, Kumar A, Kushwaha P Metallic (Inorganic) Nanoparticles: Classification, Synthesis, Mechanism, and Scope.

[155] Kumari P, Devi L, Kadian R, Waziri A, Alam MS (2024). Eco-friendly Synthesis of Azadirachta indica-based Metallic Nanoparticles for Biomedical Application & Future Prospective. Pharm Nanotechnol.

[156] Kar B, Bose A, Roy S, Chakraborty P, Chakraborty S, Das SK In Vivo and In Vitro Toxicity Study of Metallic Nanoparticles.

[157] Kumar M, Mehan N, Bhatt S, Alam MS, Gautam RK Metallic Nanoparticles for Skins and Photothermal Therapy.

[158] Darvishi M, Chekeni AM, Fazelhosseini M, Rajabalizadeh S, Rizwanullah M, Aslam M (2025). Lipid-based nanoparticles: advancing therapeutic strategies for vitiligo management. Bioimpacts.

[159] Akintelu SA, Bo Y, Folorunso AS (2020). A Review on Synthesis, Optimization, Mechanism, Characterization, and Antibacterial Application of Silver Nanoparticles Synthesized from Plants. J Chem.

[160] Yu R, Song H, Zhang X, Yang P (2005). Thermal wetting of platinum nanocrystals on silica surface. J Phys Chem B.

[161] Singhal S, Gupta M, Alam MS, Javed MN, Ansari JR Carbon Allotropes-Based Nanodevices: Graphene in Biomedical Applications.

[162] Galatage ST, Hebalkar AS, Dhobale SV, Mali OR, Kumbhar PS, Nikade SV, Kumar S, Kumar P, Pathak CS (2021). Silver Nanoparticles: Properties, Synthesis, Characterization, Applications and Future Trends. Silver Micro-Nanoparticles - Properties, Synthesis, Characterization, and Applications.

[163] Binnig G, Quate C, Gerber C (1986). Atomic force microscope. Phys Rev Lett.

[164] Binnig G, Rohrer H (1983). Scanning tunneling microscopy. Surf Sci.

[165] Wong SS, Woolley AT, Odom TW, Huang JL, Kim P, Vezenov DV (1998). Single-walled carbon nanotube probes for high-resolution nanostructure imaging. Appl Phys Lett.

[166] Cohen SH, Lightbody ML Atomic Force Microscopy/Scanning Tunneling Microscopy 3.

[167] Maeda H (2010). Tumor-selective delivery of macromolecular drugs via the EPR effect: background and future prospects. Bioconjug Chem.

[168] Fang J, Nakamura H, Maeda H (2011). The EPR effect: Unique features of tumor blood vessels for drug delivery, factors involved, and limitations and augmentation of the effect. Adv Drug Deliv Rev.

[169] Alexis F, Pridgen E, Molnar LK, Farokhzad OC (2008). Factors affecting the clearance and biodistribution of polymeric nanoparticles. Mol Pharm.

[170] Peer D, Karp JM, Hong S, Farokhzad OC, Margalit R, Langer R (2007). Nanocarriers as an emerging platform for cancer therapy. Nat Nanotechnol.

[171] Jokerst JV, Lobovkina T, Zare RN, Gambhir SS (2011). Nanoparticle PEGylation for imaging and therapy. Nanomedicine (Lond).

[172] Knop K, Hoogenboom R, Fischer D, Schubert US (2010). Poly(ethylene glycol) in drug delivery: pros and cons as well as potential alternatives. Angew Chem Int Ed Engl.

[173] Hu Q, Katti PS, Gu Z (2014). Enzyme-responsive nanomaterials for controlled drug delivery. Nanoscale.

[174] Ulbrich K, Holá K, Šubr V, Bakandritsos A, Tuček J, Zbořil R (2016). Targeted Drug Delivery with Polymers and Magnetic Nanoparticles: Covalent and Noncovalent Approaches, Release Control, and Clinical Studies. Chem Rev.

[175] Sudimack J, Lee RJ (2000). Targeted drug delivery via the folate receptor. Adv Drug Deliv Rev.

[176] Lu Y, Low PS (2002). Folate-mediated delivery of macromolecular anticancer therapeutic agents. Adv Drug Deliv Rev.

[177] Shipunova VO, Belova MM, Kotelnikova PA, Shilova ON, Mirkasymov AB, Danilova NV (2022). Photothermal Therapy with HER2-Targeted Silver Nanoparticles Leading to Cancer Remission. Pharmaceutics.

[178] Ghosh P, Han G, De M, Kim CK, Rotello VM (2008). Gold nanoparticles in delivery applications. Adv Drug Deliv Rev.

[179] Nichols JW, Bae YH (2014). EPR: Evidence and fallacy. J Control Release.

[180] Yao Y, Saw PE, Nie Y, Wong PP, Jiang L, Ye X (2019). Multifunctional sharp pH-responsive nanoparticles for targeted drug delivery and effective breast cancer therapy. J Mater Chem B.

[181] Shubayev VI, Pisanic TR 2nd, Jin S (2009). Magnetic nanoparticles for theragnostics. Adv Drug Deliv Rev.

[182] Irvine DJ, Swartz MA, Szeto GL (2013). Engineering synthetic vaccines using cues from natural immunity. Nat Mater.

[183] Kumari R, Saini AK, Kumar A, Saini RV (2020). Apoptosis induction in lung and prostate cancer cells through silver nanoparticles synthesized from Pinus roxburghii bioactive fraction. J Biol Inorg Chem.

[184] Thoidingjam S, Tiku AB (2019). Therapeutic efficacy of *Phyllanthus emblica-*coated iron oxide nanoparticles in A549 lung cancer cell line. Nanomedicine (Lond).

[185] Erdogan O, Abbak M, Demirbolat GM, Birtekocak F, Aksel M, Pasa S (2019). Green synthesis of silver nanoparticles via Cynara scolymus leaf extracts: The characterization, anticancer potential with photodynamic therapy in MCF7 cells. PLoS One.

[186] Ezhilarasi AA, Vijaya JJ, Kaviyarasu K, Maaza M, Ayeshamariam A, Kennedy LJ (2016). Green synthesis of NiO nanoparticles using Moringa oleifera extract and their biomedical applications: Cytotoxicity effect of nanoparticles against HT-29 cancer cells. J Photochem Photobiol B.

[187] Gomathi AC, Xavier Rajarathinam SR, Mohammed Sadiq A, Rajeshkumar S (2020). Anticancer activity of silver nanoparticles synthesized using aqueous fruit shell extract of *Tamarindus indica* on MCF-7 human breast cancer cell line. J Drug Deliv Sci Technol.

[188] Baharara J, Namvar F, Ramezani T, Mousavi M, Mohamad R (2015). Silver nanoparticles biosynthesized using Achillea biebersteinii flower extract: apoptosis induction in MCF-7 cells via caspase activation and regulation of Bax and Bcl-2 gene expression. Molecules.

[189] Sarkar S, Kotteeswaran V (2018). Green synthesis of silver nanoparticles from aqueous leaf extract of Pomegranate (*Punica granatum*) and their anticancer activity on human cervical cancer cells. Adv Nat Sci: Nanosci Nanotechnol.

[190] Rokade SS, Joshi KA, Mahajan K, Patil S, Tomar G, Dubal DS (2018). *Gloriosa superba* Mediated Synthesis of Platinum and Palladium Nanoparticles for Induction of Apoptosis in Breast Cancer. Bioinorg Chem Appl.

[191] Hashemi SF, Tasharrofi N, Mahmoudi Saber M (2020). Green synthesis of silver nanoparticles using Teucrium polium leaf extract and assessment of their antitumor effects against MNK45 human gastric cancer cell line. J Mol Struct.

[192] Kathiravan V, Ravi S, Ashokkumar S (2014). Synthesis of silver nanoparticles from Melia dubia leaf extract and their in vitro anticancer activity. Spectrochim Acta A Mol Biomol Spectrosc.

[193] Acharya D, Satapathy S, Yadav KK, Somu P, Mishra G (2021). Systemic Evaluation of Mechanism of Cytotoxicity in Human Colon Cancer HCT-116 Cells of Silver Nanoparticles Synthesized Using Marine Algae Ulva lactuca Extract. J Inorg Organomet Polym.

[194] (2020). Hemlata, Meena PR, Singh AP, Tejavath KK. Biosynthesis of Silver Nanoparticles Using *Cucumis prophetarum* Aqueous Leaf Extract and Their Antibacterial and Antiproliferative Activity Against Cancer Cell Lines. ACS Omega.

[195] Fehaid A, Taniguchi A (2019). Size-Dependent Effect of Silver Nanoparticles on the Tumor Necrosis Factor α-Induced DNA Damage Response. Int J Mol Sci.

[196] Venkatesan B, Subramanian V, Tumala A, Vellaichamy E (2014). Rapid synthesis of biocompatible silver nanoparticles using aqueous extract of Rosa damascena petals and evaluation of their anticancer activity. Asian Pac J Trop Med.

[197] Kanipandian N, Li D, Kannan S (2019). Induction of intrinsic apoptotic signaling pathway in A549 lung cancer cells using silver nanoparticles from *Gossypium hirsutum* and evaluation of *in vivo* toxicity. Biotechnol Rep (Amst).

[198] Venugopal K, Rather HA, Rajagopal K, Shanthi MP, Sheriff K, Illiyas M (2017). Synthesis of silver nanoparticles (Ag NPs) for anticancer activities (MCF 7 breast and A549 lung cell lines) of the crude extract of Syzygium aromaticum. J Photochem Photobiol B.

[199] Jeyaraj M, Rajesh M, Arun R, MubarakAli D, Sathishkumar G, Sivanandhan G (2013). An investigation on the cytotoxicity and caspase-mediated apoptotic effect of biologically synthesized silver nanoparticles using Podophyllum hexandrum on human cervical carcinoma cells. Colloids Surf B Biointerfaces.

[200] Meenatchi ammal R, Vijistella Bai G (2014). Green Synthesis of Silver Nanostructures Against Human Cancer Cell Lines and Certain Pathogens. Int J Pharm, Chem Biol Sci.

[201] Dey A, Manna S, Chattopadhyay S, Mondal D, Chattopadhyay D, Raj A (2019). Azadirachta indica leaves mediated green synthesized copper oxide nanoparticles induce apoptosis through activation of TNF-α and caspases signaling pathway against cancer cells. J Saudi Chem Soc.

[202] Gamal-Eldeen AM, Baghdadi HM, Afifi NS, Ismail EM, Alsanie WF, Althobaiti F (2021). Gum arabic-encapsulated gold nanoparticles modulate hypoxamiRs expression in tongue squamous cell carcinoma. Mol Cell Toxicol.

[203] Qian L, Su W, Wang Y, Dang M, Zhang W, Wang C (2019). Synthesis and characterization of gold nanoparticles from aqueous leaf extract of Alternanthera sessilis and its anticancer activity on cervical cancer cells (HeLa). Artif Cells Nanomed Biotechnol.

[204] Mittal S, Kumar C, Mallia MB, Sarma HD (2024). Re-engineered theranostic gold nanoparticles for targeting tumor hypoxia. Mater Adv.

[205] Ali EM, Abdallah BM (2025). Method of making silver nanoparticles capped with *Caralluma sinaica* extract and treatment method using the same.

[206] Patil SM, Tandon R, Tandon N (2022). Recent developments in silver nanoparticles utilized for cancer treatment and diagnosis: a patent review. Pharm Pat Anal.

[207] Abbasnezhad N, Zirak N, Shirinbayan M, Tcharkhtchi A, Bakir F (2021). On the importance of physical and mechanical properties of PLGA films during drug release. J Drug Delivery Sci Technol.

[208] Yacaman MJ, Elechiguerra JL, Burt JL, Morones JR, Larios L (2007). Glycerin based synthesis of silver nanoparticles and nanowires.

[209] Ma RH, Yu YH (2008). Nano-silver wound dressing.

[210] Todaria M, Maity D, Awasthi R (2024). Biogenic metallic nanoparticles as game-changers in targeted cancer therapy: recent innovations and prospects. Futur J Pharm Sci.

[211] Gowsalya K, Rithisa B, Shyamsivappan S, Vivek R (2024). Immune-theranostic gold nanorod-based NIR-responsive nanomedicine for the delivery of TLR7/8 adjuvant-induced effective anticancer therapy. RSC Pharm.

[212] Pornnoppadol G, Cho S, Yu JH, Kim S, Nam YS (2024). Cancer-targeting gold-decorated melanin nanoparticles for *in vivo* near-infrared photothermal therapy. Mol Syst Des Eng.

[213] Khurana D, Shaw AK, Tabassum M, Ahmed M, Shukla SK, Soni S (2023). Gold nanoblackbodies-based multifunctional nanocomposite for multimodal cancer therapy. Int J Pharm.

[214] Desai N, Chavda V, Singh TRR, Thorat ND, Vora LK (2024). Cancer Nanovaccines: Nanomaterials and Clinical Perspectives. Small.

[215] Cheng Z, Li M, Dey R, Chen Y (2021). Nanomaterials for cancer therapy: current progress and perspectives. J Hematol Oncol.

[216] Gu J, Chen F, Zheng Z, Bi L, Morovvati J, Goorani S (2023). Novel green formulation of copper nanoparticles by *Foeniculum vulgare*: Chemical characterization and determination of cytotoxicity, anti-human lung cancer and antioxidant effects. Inorg Chem Commun.

[217] Peivandi S, Dehghanzadeh H, Baghizadeh A (2022). Biosynthesis of gold nanoparticles using sansevieria plant extract and its biomedical application. Inorg Nano-Met Chem.

[218] Nazaripour E, Mousazadeh F, Doosti Moghadam M, Najafi K, Borhani F, Sarani M (2021). Biosynthesis of lead oxide and cerium oxide nanoparticles and their cytotoxic activities against colon cancer cell line. Inorg Chem Commun.

[219] Bose D, Chatterjee S (2016). Biogenic synthesis of silver nanoparticles using guava (*Psidium guajava*) leaf extract and its antibacterial activity against *Pseudomonas aeruginosa*. Appl Nanosci.

[220] Peng N, Wang Y, Ye Q, Liang L, An Y, Li Q (2016). Biocompatible cellulose-based superabsorbent hydrogels with antimicrobial activity. Carbohydr Polym.

[221] Alam MS, Javed MN, Pottoo FH, Waziri A, Almalki FA, Hasnain MS (2019). QbD approached comparison of reaction mechanism in microwave synthesized gold nanoparticles and their superior catalytic role against hazardous nitro-dye. Appl Organomet Chem.

[222] Pandit J, Alam MS, Javed MN, Waziri A, Imam SS, Shanker U, Hussain CM, Rani M (2023). Emerging Roles of Carbon Nanohorns As Sustainable Nanomaterials in Sensor, Catalyst, and Biomedical Applications. Handbook of Green and Sustainable Nanotechnology: Fundamentals, Developments and Applications.

[223] Melliti E, Mejri A, Alam MS, Ansari JR, Elfil H, Mars A MNPs for Remediation of Toxicants and Wastewater Treatment.

[224] Namita, Arti, Alam MS, Javed MN, Alam MN, Ansari JR. Catalyst Metallic Nanoparticles.

[225] Yan X, He B, Liu L, Qu G, Shi J, Hu L (2018). Antibacterial mechanism of silver nanoparticles in Pseudomonas aeruginosa: proteomics approach. Metallomics.

[226] Zhao X, Xia Y, Li Q, Ma X, Quan F, Geng C (2014). Microwave-assisted synthesis of silver nanoparticles using sodium alginate and their antibacterial activity. Colloids Surf A Physicochem Eng Asp.

[227] Velmurugan P, Lee S, Cho M, Park J, Seo S, Myung H (2014). Antibacterial activity of silver nanoparticle-coated fabric and leather against odor and skin infection causing bacteria. Appl Microbiol Biotechnol.

[228] Kora AJ, Beedu SR, Jayaraman A (2012). Size-controlled green synthesis of silver nanoparticles mediated by gum ghatti (Anogeissus latifolia) and its biological activity. Org Med Chem Lett.

[229] Rodríguez-Argüelles MC, Sieiro C, Cao R, Nasi L (2011). Chitosan and silver nanoparticles as pudding with raisins with antimicrobial properties. J Colloid Interface Sci.

[230] Siddiqui MZ, Chowdhury AR, Singh BR, Maurya S, Prasad N (2020). Synthesis, Characterization and Antimicrobial Evaluation of Piyar Gum-Induced Silver Nanoparticles. Natl Acad Sci Lett.

[231] Velusamy P, Das J, Pachaiappan R, Vaseeharan B, Pandian K (2015). Greener approach for synthesis of antibacterial silver nanoparticles using aqueous solution of neem gum (Azadirachta indica L.). Ind Crops Prod.

[232] Anavil P, Onsri P, Panprivech S, Chuenchom L, Watcharin W (2023). Green and facile synthesis of silver nanoparticles using plant extract of Aloe Barbadensis Miller and their antibacterial activity assessment. AIP Conf Proc.

[233] Sethi S, Saruchi, Medha, Thakur S, Kaith BS, Sharma N (2022). Biopolymer starch-gelatin embedded with silver nanoparticle–based hydrogel composites for antibacterial application. Biomass Conv Bioref.

[234] Narayan N, Meiyazhagan A, Vajtai R (2019). Metal Nanoparticles as Green Catalysts. Materials (Basel).

[235] Wang H, Li G, Jia L, Wang G, Tang C (2008). Controllable Preferential-Etching Synthesis and Photocatalytic Activity of Porous ZnO Nanotubes. J Phys Chem C.

[236] Royji Albeladi SS, Malik MA, Al-thabaiti SA (2020). Facile biofabrication of silver nanoparticles using Salvia officinalis leaf extract and its catalytic activity towards Congo red dye degradation. J Mater Res Technol.

[237] Akele ML, Assefa A, Madhusudhan A (2015). Microwave-Assisted Green Synthesis of Silver Nanoparticles by using Gum Acacia: Synthesis, characterization and catalytic activity studies. Int J Green Chem Bioprocess.

